# Editorial: The role of medicinal plants and natural products in modulating oxidative stress and inflammatory related disorders, Volume II

**DOI:** 10.3389/fphar.2023.1310291

**Published:** 2023-10-27

**Authors:** Gabriel A. Agbor, Jules-Roger Kuiaté, Enrico Sangiovanni, Opeolu O. Ojo

**Affiliations:** ^1^ Centre for Research on Medicinal Plants and Traditional Medicine, Institute of Medical Research and Medicinal Plants Studies, Yaoundé, Cameroon; ^2^ Research Unit of Microbiology and Antimicrobial Substances, Department of Biochemistry, Faculty of Sciences, University of Dschang, Dschang, Cameroon; ^3^ Department of Pharmacological and Biomolecular Sciences, Faculty of Pharmacy, University of Milan, Milan, Italy; ^4^ Research Institute in Healthcare Sciences, Department of Biology, Chemistry and Forensic Science, School of Sciences, Faculty of Science and Technology, University of Wolverhampton, Wolverhampton, United Kingdom

**Keywords:** antioxidants, medicinal plants, oxidative stress, inflammatory disorders, drug discovery, natural products

## Introduction

Medicinal plants and natural products have been of scientific interest for their potential role in modulating oxidative stress and inflammatory-related disorders. These resources are abundant in antioxidants, such as flavonoids, polyphenols, and vitamins which can neutralize harmful free radicals and reduce oxidative stress associated with various chronic diseases. Equally, these products can boost the immune system, thereby reducing the risk of infections and chronic inflammation. The effectiveness of various natural compounds in managing conditions such as diabetes, obesity, arthritis, cardiovascular disease, and neurodegenerative disorders has been extensively investigated. The seven articles (three reviews and four research articles) published in this Research Topic summarized some of the latest findings.

Oxidative stress is characterized by an imbalance in the rate of free radical production and the body’s ability to neutralize them with antioxidants. Free radicals are produced through processes such as the polyol pathway and the formation of advanced glycation end-products (AGEs) in hyperglycemia, and impaired mitochondrial function. The use of natural products with antioxidant potential in managing ROS generation in conditions such as hyperglycemia is currently being explored.

In this special edition, two research articles discussed the role of medicinal plants/natural products on oxidative stress in diabetes. Liu et al. reported beneficial actions of *Cordyceps militaris* (L) Fr. extracts and its active ingredient cordycepin in correcting defective glucose and lipid metabolism, as well as oxidative stress and inflammation in mice with high-fat diet- and streptozotocin-induced type 2 diabetes. The study also reported beneficial effects on gut microbiome composition and abundance. In another study, Liu et al. reported the diabetic wound healing properties of Pien-Tze-Huang (PZH), a traditional Chinese medicine in rats with streptozotocin-induced type 1 diabetes and high-fat diet-induced type 2 diabetes in mice. After topical administration and oral intubation of PTH in experimental animals, Liu et al. observed that PZH promoted wound healing characterized by enhanced re-epithelialization and vasculature in the wound tissue. The study also observed the upregulation of growth factors (VEGF-A, PDGF, and EGF) and activation of Nrf2/ARE pathway in the wound tissue of PZH-treated animals.

Many plant extracts and natural products exhibit antioxidant and inflammatory effects. Though acute and chronic inflammations are essential components of the body’s immune response to infection or injury, the body’s anti-inflammatory potential vary depending on the nature of the response. Xiao et al reported the effects of protopine (PTP), an alkaloid extracted from *Corydalis yanhusuo* W.T. Wang (Hiller et al., 1998) on mitophagy and acute lung injury in sepsis. Acute lung injury (ALI) is a severe consequence of conditions such as pneumonia, sepsis, and trauma, and is characterized by mitochondrial dysfunction and oxidative stress. PTP has been previously reported to exhibit antioxidative, anti-inflammatory, anticancer and hepatoprotective effects (Saeed et al., 1997; Nie et al., 2021). In cellular (BEAS-2B cell treated with lipopolysaccharide) and animal (mice with cecal ligation and puncture) models of sepsis, Xiao et al. reported that PTP reduced mortality, mitigated lung damage and reduced apoptosis. The study also reported the reduction in the level of expression of mitophagy-related proteins (PINK1, Parkin, LC-II) expression. In a study included in this edition, Saleem et al. reported anti-inflammatory and anti-arthritis properties of *Quercus leucotrichophora* (QL) leaf extracts. *Quercus leucotrichophora* A. camus is used traditionally in the treatment of rheumatism, joint pain, asthma, dysentery, diuretic, backache, cough, and fever (Saqib et al., 2014). Saleem et al. also reported downregulated expressions of TNF-α, IL-6, IL-1β, COX-2, and NF-κB, and upregulated expressions of IL-10, I-κB, and IL-4 in animals treated with QL extract. The study reported the presence of quercetin, gallic, sinapic, and ferulic acids in QL extracts, which have anti-inflammatory and antioxidant effects.

In this Research Topic, three review articles discussed benefits of natural products/plant extracts in treating oxidative stress-related disorders. Ngum et al. reviewed reports of natural products which modulate the expression of non-coding RNAs (ncRNAs) such as MicroRNAs (miRNAs). Certain miRNAs regulate gene expressions in oxidative stress and inflammation-associated disorders and are targets for controlling such diseases. The study reported beneficial effects of natural products such as baicalein, tanshinone IIA, geniposide, carvacrol/thymol, triptolide, oleacein, curcumin, resveratrol, solarmargine, allicin, aqueous extract or pulp of açai, quercetin, and genistein in this regard. The report however cautioned that not all natural products with antioxidant and anti-inflammatory properties modulate actions of ncRNAs. Extracts of *Zanthoxylum bungeanum* Maxim., *Canna* genus rhizome, *Fuzi-ganjiang* herb pair, *Aronia melanocarpa* (Michx) Elliot, peppermint, and gingerol were examples stated.

Another review article, Mahomoodally et al. collected data on mechanism of actions of bioactive secondary metabolites used in the management of sepsis and reported that natural products/pure compounds, including allicin, aloin, cepharanthine, chrysin, curcumin, cyanidin, gallic acid, gingerol, ginsenoside, glycyrrhizin, hesperidin, kaempferol, narciclasine, naringenin, naringin, piperine, quercetin, resveratrol, rosmarinic acid, shogaol, silymarin, sulforaphane, thymoquinone, umbelliferone, and zingerone, possess antioxidant properties and play key roles in sepsis management. The study concluded that these protective roles may be associated with the induction of endogenous antioxidant mechanisms plus the downregulation of biochemical markers in sepsis.

In the third review article, Liao et al. argued that natural products can protect the liver from drug toxicity with reference to acetaminophen. Acetaminophen is a common over-the-counter analgesic and antipyretic medication used worldwide. At high doses, the depletion of glutathione and increase in N-acetyl-p-benzoquinoneimide (NAPQI) levels have been associated with acetaminophen toxicity. Acetaminophen hepatotoxicity is also associated with oxidative stress, DNA damage, and cell necrosis (Liao et al.). The study highlighted beneficial antioxidant properties of polyphenols, terpenes, anthraquinones, and sulforaphane in hepatocytes through the nuclear factor erythroid 2–related factor 2 (Nrf2) pathway.

## Conclusion

Natural products play important role in the management of oxidative stress-related disorders, as summarized in the seven articles published in this edition. However, it is important to note that while secondary metabolites show promise in preclinical studies and have demonstrated some potential benefits, their use in the management of diabetic sores and sepsis requires special care and should be administered under medical supervision. Medicinal plants should not replace conventional medical treatment for either diabetic sores or sepsis due of the complex nature of their management.
